# *Micrococcus lylae* MW407006 Pigment: Production, Optimization, Nano-Pigment Synthesis, and Biological Activities

**DOI:** 10.3390/biology11081171

**Published:** 2022-08-04

**Authors:** Yahya H. Shahin, Bassma H. Elwakil, Doaa A. Ghareeb, Zakia A. Olama

**Affiliations:** 1 Department of Medical Laboratory Technology, Faculty of Applied Health Sciences Technology, Pharos University in Alexandria, Alexandria 21648, Egypt; 2 Department of Botany and Microbiology, Faculty of Science, Alexandria University, Alexandria 21526, Egypt; 3 Biological Screening and Preclinical Trial Lab, Biochemistry Department, Faculty of Science, Alexandria University, Alexandria 21526, Egypt

**Keywords:** microbial pigment, statistical optimization, antimicrobial, anticancer, nano-pigment

## Abstract

**Simple Summary:**

The global crisis of increased mortality rates due to the emergence of antimicrobial resistance and cancers has increased researchers’ efforts to find new, potent solutions through implementing natural products in the pharmaceutical industry. The present investigation produced echinenone (yellowish-orange pigment) from *Micrococcus lylae* MW407006 with potent pharmacological activities. A response surface methodology statistical design was used to optimize the biomass production, pigment concentration, and antimicrobial activity. The Spearman correlation coefficient was assessed, which indicated a strong linear relationship between biomass production, pigment concentration, and antimicrobial activity. Nano-echinenone was physically synthesized through the ball-milling technique. The synthesized nano-echinenone showed higher pharmacological activities (antimicrobial, antioxidant, and antitumor activities) in comparison with the crude pigment. The significantly high selectivity index of the synthesized nano-echinenone proved its safety and paved the way for its possible use in the pharmaceutical industry.

**Abstract:**

Bacterial pigments (e.g., melanin and carotenoids) are considered to be among the most important secondary metabolites due to their various pharmacological activities against cancer and microbial resistance. Different pigmented bacterial strains were isolated from soil samples from El Mahmoudiyah governance and screened for their antimicrobial activity. The most promising pigment producer was identified as *Micrococcus lylae* MW407006; furthermore, the produced pigment was identified as echinenone (β-carotene pigment). The pigment production was optimized through a central composite statistical design to maximize the biomass production, pigment concentration, and the antimicrobial activity. It was revealed that the most significant fermentation parameters were the glucose (as a carbon source) and asparagine (as a nitrogen source) concentrations. Nano-echinenone was synthesized using the ball milling technique, characterized, and finally assessed for potential antimicrobial, antioxidant, and antitumor activities. The data revealed that the synthesized nano-echinenone had higher antimicrobial activity than the crude pigment. The cytotoxic potency of echinenone and nano-echinenone was investigated in different cell lines (normal and cancer cells). The inhibition of cell proliferation and induction of cell death was observed in Caco-2 and Hep-G2 cells. The data proved that nano-echinenone is a suitable candidate for use as a safe antimicrobial and anti-hepatocellular-carcinoma agent.

## 1. Introduction

Microbial pigments have gained the interest of more researchers recently, not only due to their utilization as colorants in industries such as food, plastic, textile, paint, printing, and paper, but also because they possess several pharmacological properties, such as antimicrobial, antioxidant, antileishmanial, anticancer, antiviral, and various other bio-pharmacological activities, with have proven their potentiality and efficacy in various pharmaceutical applications [1].

Bacteria were considered as the ideal pigment-producing source because of several advantages, namely the short and rapid life cycle, easy maintenance or preservation of bacterial cultures, scalability for industrial use, the ability to adapt to environmental changes, the ability to grow on cheap, easily available media, simple extraction techniques, and renewable sources that can reduce the pigment production cost [2]. 

Pigmented bacteria can be pervasive and prevalent in different niches, such as the freshwater, soil, and marine environments [3]. Carotenoids may function as vitamin supplements and oxidative stress-protecting agents, and prevent photo-aging and sunburns. Epidemiological studies have shown that people with high β-carotene intake have a reduced risk of lung cancer [3]. The most prevalent pigment-producing bacteria include *Streptomyces* spp., *Arthrobacter* spp., *Pseudomonas* spp., *Staphylococcus* spp., *Micrococcus* spp., and *Chryseobacterium* spp. with diverse pigment production, including carotenoids, flexirubin, pyocyanin, prodigiosin, violacein, staphyloxanthin, flavonoids, melanin, etc. [2,4]. 

The optimization of cultural environmental factors has also been outlined for enhancing the pigment yield in *Duganella* spp. [5] and *Bacillus* spp. [6]. Statistical optimization experimental designs, such as response surface methodology (RSM), can be applied to collectively optimize the environmental and nutritional factors. RSM combines statistical and regression analyses, which help in building models, to study the interactions between the tested variables and finally select the optimum variable(s) conditions [7]. 

On the other hand, nanotechnology is currently employed as a tool to explore the darkest avenues of medical science in several ways, namely imaging, sensors, targeted drug delivery and gene delivery systems, and artificial implants. New-era drugs are nanoparticles of polymers, metals, or ceramics that can combat medical conditions, such as cancer, and fight several types of human pathogens. Ball milling is a mechanical process that enables the purposeful execution of physical and chemical transformations in powdered materials. The method is based on evidence that shows that the physical and chemical behaviors of individual molecules, and ordered and disordered solids, can be affected by non-hydrostatic mechanical stresses and the associated strains [8]. Ball milling is usually performed at room temperature, which has the undeniable advantages of lowering the limitations of using high temperatures, toxic solvents, and challenging in situ polymerization procedures. Additionally, ball milling offers an intriguing option for industrial manufacturers to efficiently synthesize nanoparticles (especially for temperature-sensitive molecules) in a cost-effective way [9].

The aim of the present work was to isolate a promising bacterial pigment producer and evaluate the potential antimicrobial, antioxidant, and antitumor activities of the potent pigment. Then, a novel nano-pigment was synthesized using the ball-milling technique to sustain the concept of cost-effective large-scale production. The antimicrobial, antioxidant, and antitumor activities were compared between the pigment in its conventional form and that in its nano-form.

## 2. Materials and Methods

### 2.1. Microorganisms

Different bacterial and fungal multi-drug-resistant (MDR) pathogens were used throughout the present work, namely Methicillin-resistant *Staphylococcus aureus* (MRSA), *Staphylococcus aureus*, *Escherichia coli*, *Candida albicans*, *Klebsiella pneumoniae, Pseudomonas aeruginosa*, *Listeria monocytogenes*, *Salmonella Typhi, Proteus vulgaris*, and *Acinetobacter baumannii*. All the strains were kindly provided and identified by the Surveillance Microbiology Department’s strain bank of Al-Shatby Pediatric Hospital, Alexandria.

### 2.2. Pigment Producing Bacteria 

#### 2.2.1. Isolation 

Different soil samples were collected under sterile conditions from El Mahmoudiyah governance (30.031608° N 31.257721° E). The soil samples were serially diluted and cultivated on nutrient agar (NA) (Merck Co., NJ, USA) for the isolation of pigmented colonies. Colored bacterial colonies were selected and maintained as pure cultures in NA slants with periodic transfer to fresh media.

#### 2.2.2. Pigment Extraction 

The selected bacterial pigment producers were grown in nutrient broth under shaking (150 rpm/min) for 48 h at 35 ± 2 °C. After incubation, the colored bacterial suspension was centrifuged for 15 min at 5000× *g*. Then, the pigment was extracted with chloroform (chloroform:supernatant = 1:2, *v/v*). After two hours, the organic phase was mixed with 0.2 mL of HCl (1:1, *v*/*v*), and the extract was obtained after evaporation to dryness, followed by the re-dissolution of each extracted pigment (0.2 mg) in 1 mL of dimethyl sulfoxide (DMSO) for further analyses [10].

#### 2.2.3. Screening for Antimicrobial Activity

DMSO-dissolved pigments were assessed for their antimicrobial effect against different multi-drug-resistant (MDR) pathogens, namely *Klebsiella pneumoniae, Escherichia coli*, *Proteus vulgaris*, *Pseudomonas aeruginosa*, *Salmonella Typhi*, *Listeria monocytogenes*, *Acinetobacter baumannii*, *Staphylococcus aureus*, and *Candida albicans* using the disc-diffusion method according to Afifi et al. [11]. Isolated pigments with the highest potential for antimicrobial activity were selected for further investigation. The susceptibility profile of the tested pathogens to some commonly known antibiotics is provided in Table S1.

#### 2.2.4. Identification of the Most Potent Pigment Producer

The most promising bacterial pigment producer with the highest antimicrobial activity was subjected to identification using morphological, biochemical, and 16S rRNA sequencing. The bacterial DNA sample was amplified using forward (5′-AGTTTGATCCTGGCTCAG-3′) and reverse (5′-GGCTTACCTTGTTACGACTT-3′) 16S rRNA primers. After sequencing the 16S rDNA, a multiple sequence alignment was performed in accordance with the National Center for Biotechnology Information (NCBI) database. Finally, the phylogenetic tree of the promising isolate was generated through distance matrix analysis using the NT system [12].

#### 2.2.5. Liquid Chromatography–Mass Spectrometry (LC-MS), Nuclear Magnetic Resonance (NMR), and Fourier-Transform Infrared (FTIR) Spectroscopic Analysis

Pigment characterization was conducted using liquid chromatography–mass spectrometry (LC-MS), NMR, and FTIR spectroscopy. The NMR spectra were obtained using Bruker spectrometers (500 and 400 MHz). The FTIR spectrum of the extracted pigments was analyzed using a Perkin–Elmer R79521 (Perkin Elmer, Inc., Waltham, MA, USA)). FTIR spectroscopy (Perkin Elmer, Inc., Waltham, MA, USA) was conducted with a 2 cm^−1^ resolution and wavenumber of 4000 cm^−1^ to 450 cm^−1^ in sixty-four scans. On the other hand, LC-MS analysis (LCMS-8060NX, Shimadzu Co., Kyoto, Japan) was conducted using a C-18 octadecylsilane column with a dual λ UV detector and binary pump. The fractions eluted at different time intervals from LC were further subjected to mass spectrometry (MS) analysis. MS analysis of the samples was conducted over a mass range of 50–1500 m/z [13].

### 2.3. Optimization of the Cell Biomass Concentration, Pigment Production, and Antimicrobial Activity

#### 2.3.1. Optimization Using One Factor at a Time

In a trial to select the most promising carbon and nitrogen sources that lead to maximum antimicrobial activity, different carbon sources (glucose, sucrose, lactose, mannose, fructose, and glycerol) and nitrogen sources (peptone, tryptone, casein, yeast, and asparagine) were evaluated one at a time. Pigment was extracted periodically at 24 h intervals throughout a 6 d incubation period and analyzed for antimicrobial activity using the disc-diffusion method. The best optimized carbon and nitrogen source was selected for further experiments [14].

#### 2.3.2. Optimization Using Central Composite Design (Response Surface Methodology)

A central composite design (CCD) was applied for studying and optimizing the most effective variables that affected the antimicrobial activity, biomass production, and pigment concentration. Moreover, interactions between multiple parameters were evaluated by employing statistical modeling with response surface methodology (RSM), which uses a statistical approach with five levels (−2.37841, −1, 0, 1, and 2.37841) to assess the effect of the conditions’ variations in different parameters, to detect the optimal conditions (Table 1). The environmental and nutritional factors of the most promising isolate, namely the carbon source level, nitrogen source level, pH, temperature, and incubation time, were optimized to maximize the antimicrobial activity of the extracted pigment (response R1), the biomass production concentration (response R2), and the pigment concentration (response R3) [15]. The mathematical relationship of the response of these parameters can be illustrated by a quadratic (second degree) polynomial equation (Equation (1)), where y is the response value; b_0_ is the constant; x_1_, x_2_, x_3_, x_4_, and x_5_ are the independent parameters; b_1_, b_2_, b_3_, b_4_, and b_5_ are the linear coefficients; b_12_, b_13_, b_14_, b_15_, b_23_, b_24_, b_25_, b_34_, b_35_, and b_45_ are the cross-product coefficients; and b_11_, b_22_, b_33_, b_44_, and b_55_ are the quadratic coefficients. A total of fifty runs were processed to estimate the coefficients of the model using multiple linear regressions. The design of the experiments was conducted using Design-Expert 12.0^®^.

(1) $$\begin{array}{ll} y=b_{0}+b_{1}x_{1}+ & b_{2}x_{2}+b_{3}x_{3}+b_{4}x_{4}+b_{5}x_{5}+b_{11}x_{1}^{2}+b_{22}x_{2}^{2}+b_{33}x_{3}^{2}+b_{44}x_{4}^{2}\\ & +b_{55}x_{5}^{2}+b_{12}x_{1}x_{2}+b_{13}x_{1}x_{3}+b_{14}x_{1}x_{4}+b_{15}x_{1}x_{5}+b_{23}x_{2}x_{3}\\ & +b_{24}x_{2}x_{4}+b_{25}x_{2}x_{5}+b_{34}x_{3}x_{4}+b_{35}x_{3}x_{5}+b_{45}x_{4}x_{5} \end{array}$$

### 2.4. Kinetic Study and Pigment Yield Calculation

A kinetic study of the cell growth (biomass) was conducted and the pigment production of the most potent strain was evaluated while fixing the optimized conditions. At specific time intervals, 100 mL of the medium was harvested and centrifuged at 20,000× *g* for 20 min. The crude pigment was extracted and weighed. Both the cell mass and extracted pigment were converted to g/L and plotted against time. The specific pigment production and the specific growth rate were calculated according to the following equations (Equations (2) and (3)):

(2) μX=(dX|dt)

where μ is the specific growth rate, X is the cell biomass concentration (g/L), and t is the specific time (h).

(3) PX=(dP|dt)

where P is the concentration of the extracted pigment (g/L) at time t (d) and X is the cell biomass concentration (g/L) [2].

### 2.5. Synthesis of the Nano-Pigment Using the Ball Milling Technique 

The optimized extracted pigments were introduced to the ball mill for nanosized pigment fabrication. Physical synthesis experiments were performed with a FRITSCH planetary ball mill (Pulverisette 7 premium line, two hardened steel vials, 80 cm^3^ volume, and charged with 60 hardened steel balls that were 3 mm in nominal diameter as the milling bodies). Two grams of the extracted pigments were used as the precursor material. The angular velocity of the milling was fixed at 200 rpm for 30 min [16].

#### 2.5.1. Physicochemical Characterization of the Synthesized Nanoparticles 

The dynamic light scattering (DLS) technique was used to evaluate the Zeta potential, particle size (PS), and polydispersity index (PDI) of the synthesized nanoparticles using a Malvern Zetasizer according to Elnaggar et al. [17]. The FTIR spectrum of the synthesized nanoparticles was also analyzed using Perkin–Elmer R79521 FTIR (USA). The shape, size, and ultra-structure of the synthesized nanoparticles were examined using a transmission electron microscope (TEM) (JEM-100 CX, JOEL, Tokyo, Japan) (resolution 3 nm at 30 kV) [18].

#### 2.5.2. Antimicrobial Activity of the Synthesized Nanoparticles

The synthesized nanoparticles were evaluated for antimicrobial activity against different pathogens using the disc-diffusion method and minimum inhibitory concentration (MIC). The bacterial lethality curve was also assessed and a transmission electron microscopy study of the microbial treated cells was conducted [17].

#### 2.5.3. Antioxidant Activity 

The free-radical scavenging activity of the extracted pigments and the prepared nano-pigment was measured by 1,1-diphenyl-2-picrylhydrazyl (DPPH). Amounts of 2.5 mL of the pigment and different concentrations of the prepared nano-pigments were mixed with 1.0 mL of a DPPH methanolic solution individually. The mixture was shaken vigorously and then left at room temperature for 30 min in the dark. The absorbance of the resulting solution was then measured at 518 nm. The scavenging activities of the extracted pigment were evaluated according to the following equation (Equation (4)):

(4) $$\mathrm{Scavenging}\;\mathrm{activity}=\frac{\mathrm{Abs}\;\mathrm{DPPH}-\mathrm{Abs}\;S}{\mathrm{Abs}\;\mathrm{DPPH}}\times 100$$

where Abs S is the absorbance of the solution when the sample (pigment and nano-pigment) was added at a particular concentration, while Abs DPPH is the absorbance of the DPPH solution. The tested concentration providing 50% inhibition (EC50) was calculated from the graph of the scavenging effect percentage against different concentrations [19].

#### 2.5.4. Cytotoxicity Assay Using Human Cancer Cell Lines

Different concentrations of the selected pigment and nano-pigments were used to examine their effect on different cell lines. In a 96-well tissue culture plate, Vero, WI38, Caco-2, and Hep-G2 cells (cell lines purchased from ATCC, CCL-81, CCL-75, HTB-37, and HB-8065, respectively) were plated each in their respective culture media at a density of 5000 cells/well. The color intensity was measured at 490 nm in a microtiter plate-reader spectrophotometer. Using the relation between the used concentrations and neutral red intensity value, the IC50 of each compound was calculated [20].

Moreover, the selectivity index was calculated as follows (Equation (5)):

(5) $$\mathrm{Selectivity}\;\mathrm{index}\;=\frac{\mathrm{IC}50\;\mathrm{of}\;\mathrm{normal}\;\mathrm{cell}}{\mathrm{IC}50\;\mathrm{of}\;\mathrm{cancer}\;\mathrm{cells}}$$

#### 2.5.5. Determination of Cellular Reactive Oxygen Species (ROS)

Serial concentrations of the synthesized nano-pigment were incubated with HepG-2 and Caco-2 one at a time for 72 h, and the cellular ROS level was quantified with 5 μM of 2,7-dichlorofluorescin diacetate (DCFH-DA) after incubation for 30 min at 37 °C. The fluorescence intensity (at 488 nm) and emission (at 530 nm) of the oxidized form of DCFH-DA were detected after washing the cells [21,22].

#### 2.5.6. Relative Change in the Genetic Expression of Proapoptotic (p53 and BAX) and Anti-Apoptotic (Bcl2) Genes 

The total RNA was extracted from the control and nano-pigment-treated HepG-2 and Caco-2 cells. Then, cDNA was synthesized using Gene JET cDNA synthesis kits (Thermo Scientific, Waltham, MA, USA). Real-time PCR (RT-PCR) was conducted using SYBR green master mix and the following specific primers (Forward/Reverse): 5′-ATGTTTTGCCAACTGGCCAAG-3′/5′-TGAGCAGCGCTCATGGTG-3′, 5′-CCGCCGTGGACACAGAC-3′/5′-AGAAAACATGTCAGCTGCCA-3′, and 5′-TCCGATCAGGAAGGCTAGAGTT-3′/5′-TCGGTCTCCTAAAAGCAGGC-3′ for p53, BAX, and Bcl2 genes, respectively. The relative gene expression was calculated by using the rule of 2^−∆∆CT^ [21,23].

### 2.6. Statistical Analyses

The Design-Expert 12.0^®^ software package from Stat-Ease was used to implement the design of the experiments using multifactorial Central Composite Design (CCD). Fisher’s Least Significant Difference (LSD) post hoc test was applied after running Analysis of Variance (ANOVA) to analyze and identify the pairs of means that were statistically different according to the t-values. The results were the means of three trials. The means of the treatments were considered significant when *p* < 0.05. A Spearman correlation test was used to measure the possible correlation, strength, and direction between each response (the closer the absolute value of the coefficient to one, the stronger the relationship between the tested responses).

## 3. Results and Discussions 

### 3.1. Isolation of Pigment-Producing Bacteria 

Different pigment-producing bacteria (six isolates) were selected from different soil samples collected at El Mahmoudiyah governance. All the pigment-producing isolates were Gram-positive bacilli and cocci with yellow, orange, and red pigments. 

### 3.2. Antimicrobial Activity of the Extracted Pigments

A screening study for the antimicrobial activity of the extracted pigments revealed that isolate numbers 1, 3, and 6 were the most promising pigment producers (Table 2). The highest inhibition zone diameter was observed with the extracted pigment of isolate number 3 against *Pseudomonas aeruginosa* and *Staphylococcus aureus* (12 mm diameter). The extracted pigment of isolate 3 (yellowish orange) had the highest antimicrobial activity against all of the tested strains; hence, it was chosen for further analyses. 

Goswami et al. [24] also isolated pigment-producing bacteria from soil samples, and the most prevalent pigment was yellow, while the isolated bacteria were Gram-positive cocci. Zohari et al. [25] isolated 104 isolates from soil and water samples, and only 5 strains were pigment (carotenoid) producers (70% *M. luteus* and 30% *Micrococcus roseus*). Qayyum et al. [10] reported that red pigments from *Serratia marcesens* had antibacterial activity against *Staphylococcus aureus* and *E. coli*, while yellow pigments from *Actinomyces* spp. had antibacterial activity against *Salmonella* spp. and *Staphylococcus aureus.*

### 3.3. Identification of the Most Promising Isolate

**Figure 1:**
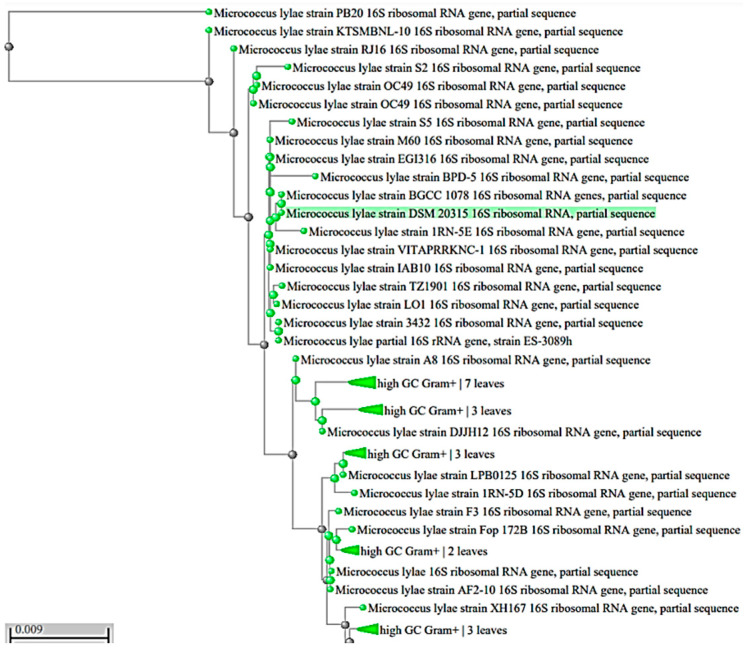
Phylogenetic tree of the isolated pigmented strains based on the 16S rRNA gene sequence. The highlighted green color indicates the identified bacterial strain.

The morphological and biochemical observations of the most potent isolate (isolate no. 3) showed that the colonies were circular, entire, and convex with a bright orange–yellow pigment. Isolate no. 3 was a Gram-positive, cocci, catalase, oxidase-positive, and urease-negative bacterium. Then, 16S rRNA sequencing followed by a multiple sequence alignment was performed in accordance with the National Center for Biotechnology Information (NCBI) database. The promising strain was identified as *Micrococcus lylae* with accession number MW407006 (Figure 1). 

### 3.4. Pigment Identification and Characterization 

**Figure 2:**
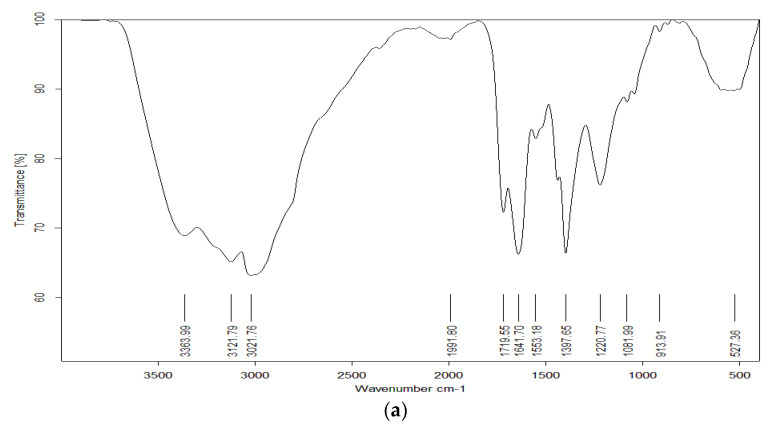

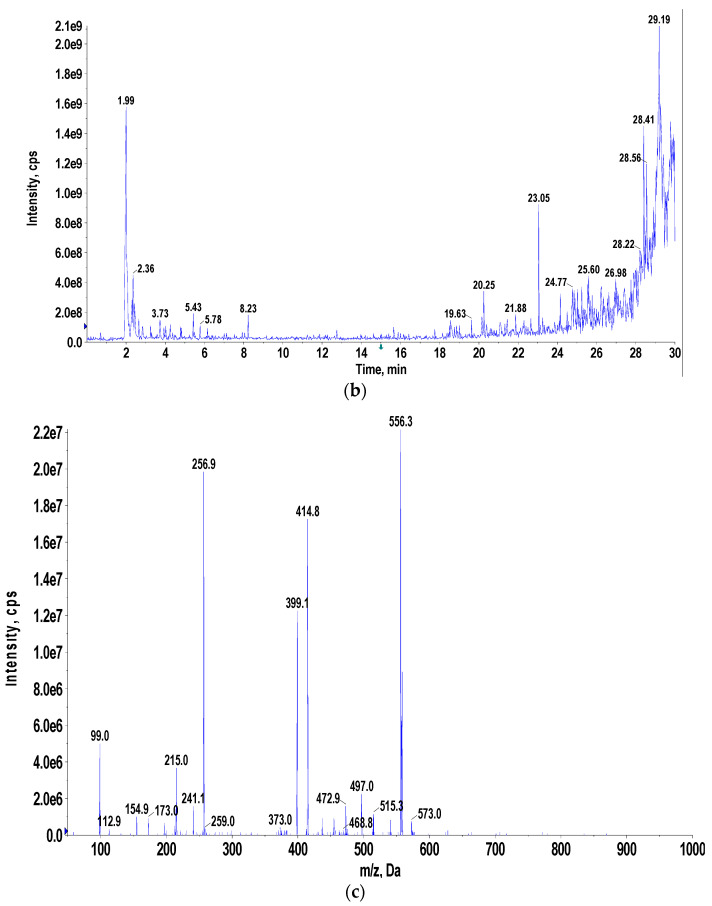
Crude pigment characterization of *Micrococcus lylae* MW407006: (**a**) FTIR, (**b**) LC/MS, and (**c**) MS/MS spectra of the extracted pigment (Echinenone) at 8.23 min.

FTIR and LC/MS analyses revealed that the *Micrococcus lylae* MW407006 pigment was echinenone (Figure 2a,b) when compared with the MassBank database. The most abundant ion [M+H]^+^ was 556.3 m/z. The ^1^H-NMR spectra of the extracted pigment were explained according to Wang et al. [26], where the resonance of protons attached to methyl was observed between 1 and 2 ppm, while the proton attached to the ring was observed between 1.5 and 2.5 ppm (Figure S1). The yellow–orange carotenoid protein has a ketocarotenoid molecule, 3′-hydroxyechinenone (hECN), that spans its N-terminal and C-terminal domains. The ν_1_ band, around 1520 cm^−1^, resulted from the stretching vibrations of C=C double bonds. The frequency of the ν_1_ band depends on the length of the conjugated chain of the π-electron and on the molecular configuration of the carotenoid. The ν_2_ band at 1160 cm^−1^ was observed due to the stretching vibrations of C–C single bonds coupled with C–H in the in-plane bending mode—this region is considered as a fingerprint for the carotenoid configuration assignment, i.e., isomerization states [27]. Altogether, these spectra are extremely similar to those of β-carotene or of any carotenoid, especially the conjugated carbonyl groups. The presence of a carbonyl group in the echinenone molecular structure is the only chemical difference between it and β-carotene. Recently, it was confirmed that the frequency of *ν*_1_ of carotenoid molecules exhibits low, but significant, sensitivity to the polarizability of the surrounding solvent around the molecules [28]. The echinenone spectra models confirmed that the ν_1_ band for these carotenoid molecules corresponds to a C=C stretching delocalized over the whole conjugated chain, but with higher participation of the central bonds [29].

### 3.5. Optimization of the Pigment Production, Biomass Concentration, and Antimicrobial Activity 

#### 3.5.1. Optimization Using One Factor at a Time:

**Figure 3:**
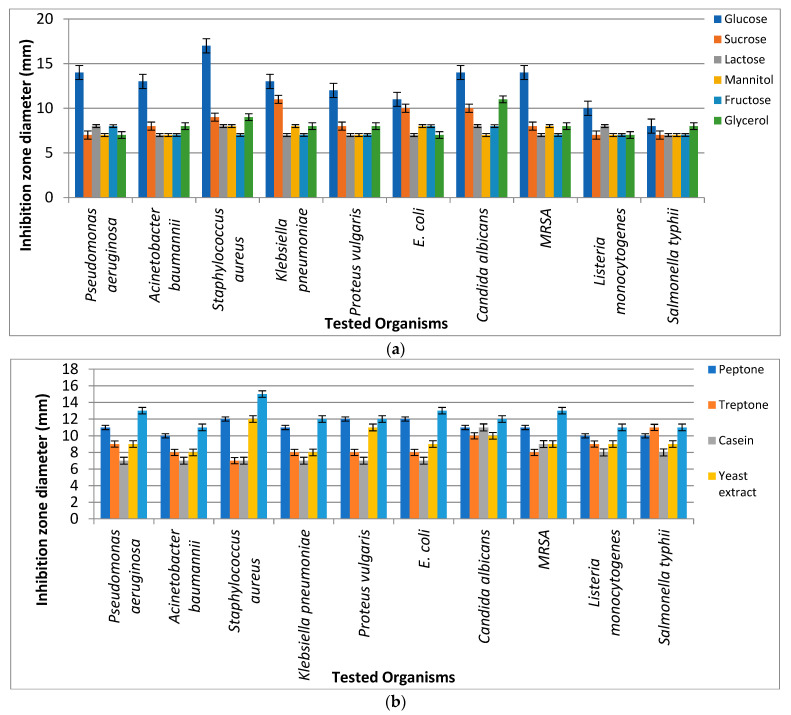
Antibacterial activity of *Micrococcus lylae* (MW407006) pigment as affected by (**a**) carbon and (**b**) nitrogen sources.

Different fermentation parameters (namely carbon and nitrogen sources) were used to select the most suitable source that led to the highest antimicrobial activity of pigments extracted from *Micrococcus lylae* MW407006. It was revealed that the most suitable carbon and nitrogen sources were glucose and asparagine, respectively, which showed the highest inhibition zone diameters against the tested pathogens (Figure 3).

#### 3.5.2. Central Composite Design (CCD) Optimization of *Micrococcus lylae* (MW407006) 

**Figure 4:**
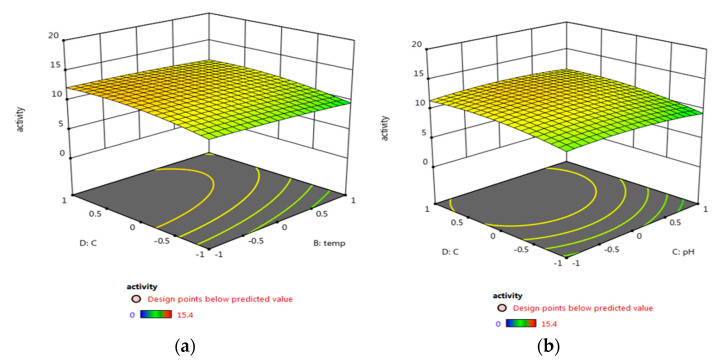

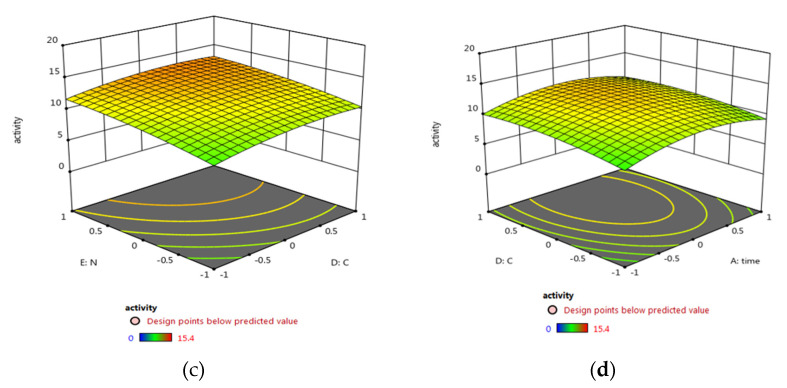
Three-dimensional surface plots for the effect of the tested parameters interactions that led to maximum antimicrobial activity (R1): (**a**) carbon source and temperature, (**b**) carbon source and pH, (**c**) carbon source and nitrogen source, and (**d**) carbon source and incubation time.

**Figure 5:**
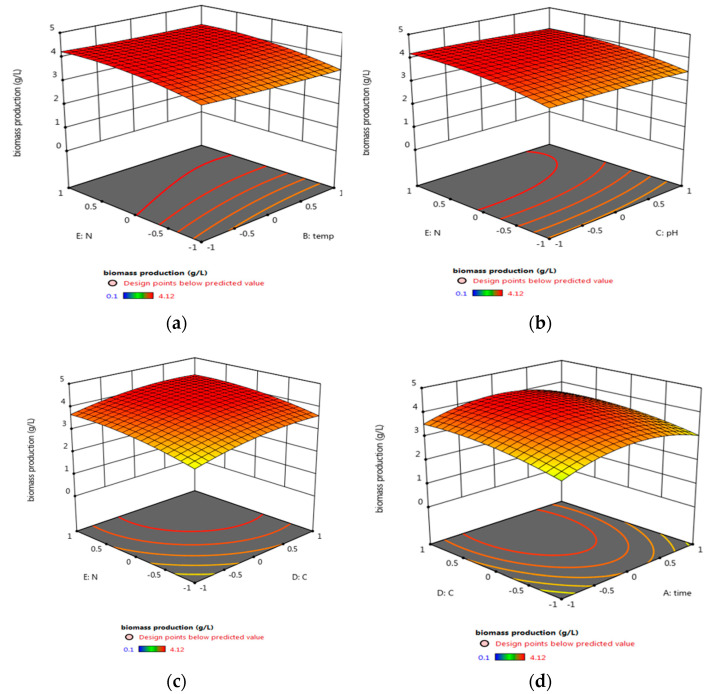
Three-dimensional surface plots for the effect of the tested parameters’ interactions that lead to the maximum biomass production concentration (R2): (**a**) nitrogen source and temperature, (**b**) nitrogen source and pH, (**c**) carbon source and nitrogen source, and (**d**) nitrogen source and incubation time.

**Figure 6:**
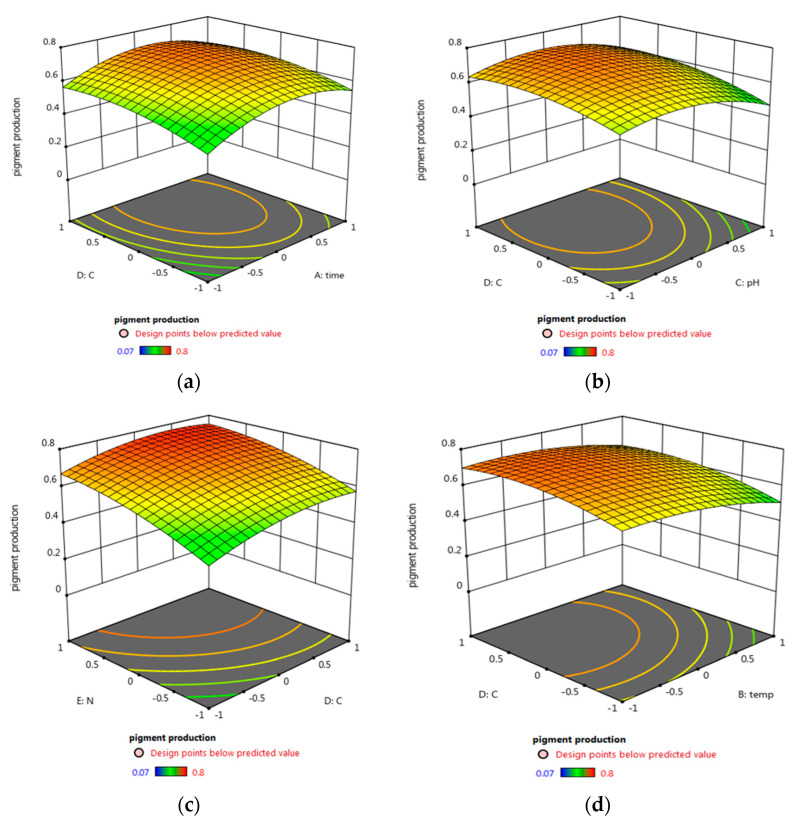
Three-dimensional surface plots for the effects of the tested parameter interactions that lead to maximum pigment production (R3): (**a**) carbon source and incubation time, (**b**) carbon source and pH, (**c**) carbon source and nitrogen source, and (**d**) carbon source and temperature.

In order to maximize the antimicrobial activity, biomass, and pigment production, a central composite design was used. Different fermentation factors were applied in five levels using the Response Surface Methodology (RSM); each variable was studied at five coded levels (−2, −1, 0, 1, and 2) for deeper analysis and higher efficiency of the biomass, pigment production, and antimicrobial activity (Table 1). The investigation was conducted across five factors using RSM, and their effect was statistically analyzed using Design-Expert 12.0^®^ (Table 3, Figure 4). Factors with significant effects on the antimicrobial activity (R1), biomass (R2), and pigment (R3) concentrations are shown in Figure 4, Figure 5 and Figure 6, respectively. The model fitting in the form of Analysis of Variance (ANOVA) (Tables S2 and S3) along with the design integrity and precision were analyzed (Equations (6)–(8)). The significance of each coefficient was determined by Fisher’s F-test and *p*-values. It was revealed that trial 20 showed the highest antimicrobial activity, as well as the maximum biomass and pigment production concentration (Table 3). The optimum condition for the potent strain *Micrococcus lylae* MW407006 was 0.5% carbon and 0.5% nitrogen sources with an initial pH of 7 for 4 days of incubation at 25 °C. This was implied by the linear effects of the carbon source concentration and nitrogen source concentration, as well as the significant quadratic effect of time and the carbon source concentration (*p* ≤ 0.05) to maximize the antimicrobial activity (R1). On the other hand, the maximum biomass production (R2) was determined by the linear effects of the carbon source and nitrogen source concentrations, as well as the quadratic effects of both the carbon and nitrogen source concentrations that were noted as significant (*p* ≤ 0.05). Moreover, the maximum pigment production (R3) was implied by the linear effects of the carbon and nitrogen source concentrations, as well as the quadratic effects of the incubation time, pH, and carbon source concentration that were significant (*p* ≤ 0.05). The remaining interaction terms were insignificant (*p* > 0.05) (Table S2). The Spearman correlation coefficient indicated a significantly strong linear relationship between pigment/biomass production (0.99). Moreover, a strong linear relationship was also noticed (reaching 0.92) for both pigment production/antimicrobial activity and biomass production/antimicrobial activity.

(6) $$\begin{array}{l} \mathrm{Antimicrobial}\;\mathrm{Activity}\\ \;\;\;\;\;\;\;\;=12.215+0.40A-0.44B-0.31C+0.81D+1.41E\\ \;\;\;\;\;\;\;\;-0.14\mathrm{AY}+0.21\mathrm{AC}+0.08\mathrm{AD}+0.01\mathrm{AE}-0.38\mathrm{BC}+0.06\mathrm{BD}\\ \;\;\;\;\;\;\;\;+0.07\mathrm{BE}+0.17\mathrm{CD}-0.33\mathrm{CE}-0.10\mathrm{DE}-1.89\mathrm{AA}-0.18\mathrm{BB}\\ \;\;\;\;\;\;\;\;-0.58\mathrm{CC}-0.77\mathrm{DD}-0.46\mathrm{EE} \end{array}$$

(7) $$\begin{array}{l} \mathrm{Biomass}\;\mathrm{production}\\ \;\;\;\;\;\;\;=4.035+0.04A-0.07B-0.07C+0.28D+0.30E\\ \;\;\;\;\;\;\;-0.048\mathrm{AY}+0.044\mathrm{AC}+0.024\mathrm{AD}+0.018\mathrm{AE}-0.053\mathrm{BC}\\ \;\;\;\;\;\;\;+0.016\mathrm{BD}+0.022\mathrm{BE}+0.033\mathrm{CD}-0.018\mathrm{CE}-0.022\mathrm{DE}\\ \;\;\;\;\;\;\;-0.55\mathrm{AA}-0.038\mathrm{BB}-0.042\mathrm{CC}-0.21\mathrm{DD}-0.19\mathrm{EE} \end{array}$$

(8) $$\begin{array}{l} \mathrm{Pigment}\;\mathrm{production}\\ \;\;\;\;\;\;\;\;=0.70+0.04A-0.04B-0.031C+0.05D+0.097E\\ \;\;\;\;\;\;\;\;-0.013\mathrm{AY}+0.021\mathrm{AC}+0.009\mathrm{AD}+0.01\mathrm{AE}-0.038\mathrm{BC}\\ \;\;\;\;\;\;\;\;+0.007\mathrm{BD}+0.007\mathrm{BE}+0.017\mathrm{CD}-0.03\mathrm{CE}-0.01\mathrm{DE}\\ \;\;\;\;\;\;\;\;-0.102\mathrm{AA}-0.039\mathrm{BB}-0.079\mathrm{CC}-0.047\mathrm{DD}-0.038\mathrm{EE} \end{array}$$

where A is the independent variable of time, B is the temperature, C is the pH, D is the carbon source, and E is the nitrogen source. 

A similar study was conducted by Zohari et al. [30] on the optimization of carotenoid production by *Micrococcus* spp. They mentioned that the optimum growth and pigment production conditions were 1% carbon and 2% nitrogen source with an initial pH of 7 for 4 days of incubation at 25 °C. This result may explain that the observed antimicrobial activity in the present study was due to the high carotenoid content. Fariq et al. [31] studied the effect of different fermentation parameters on carotenoid production and activity through a RSM study. It was revealed that the most sensitive parameters for *Salinicoccus sesuvii* MB597 were the incubation temperature and the initial pH, while *Aquisalibacillus elongatus* MB592 and *Halomonas aquamarina* MB598 were only influenced by the incubation temperature. Bhosale [32] suggested that carotenoid production by the organism was mainly dependent on the environmental conditions in line with the culture media composition. It was also emphasized that, by decreasing the incubation temperature, an increase in the carotenoid concentration was observed.

### 3.6. Antimicrobial Investigations of the Optimized Pigment

#### MIC, MBC, and MIC Index

*Pseudomonas aeruginosa, Staphylococcus aureus*, and *Candida albicans* were the most sensitive strains; hence, they were chosen for further analyses. The optimized pigment showed a bactericidal effect against the tested strains (MIC index > 4). The highest antibacterial effect was recorded against *Staphylococcus aureus*, where the MIC value reached 16 μg/mL (Table 4). Rashid et al. [33] obtained different soil samples and isolated several pigmented bacteria with antibacterial effects (MIC value ranged from 1500 to 4000 μg/mL against Gram-positive and Gram-negative bacteria). This implies that further exploration of different soil samples under different environmental conditions may pave the way to explore more pigmented bacteria with potential pharmacological activities. 

### 3.7. Growth Kinetics in Relation to the Pigment Yield

**Figure 7:**
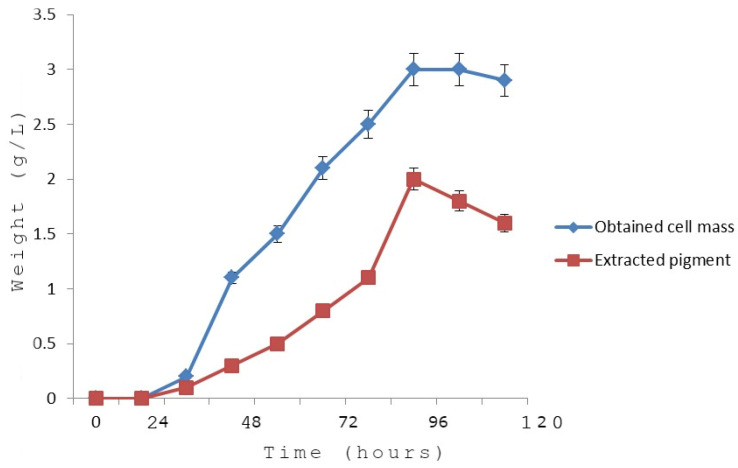
Growth kinetics of *Micrococcus lylae MW407006* and pigment production kinetics.

The growth kinetics curve of the obtained cell mass versus time (Figure 7) proved that the log (exponential) phase continued for 40 h (started after 15 h and continued until 55 h post-inoculation); then, the stationary phase was recorded up to 82 h; finally, the bacterial cells entered the decline (death) phase. It was noted from the kinetic study that the pigment production started in the log phase and peaked at the stationary phase (between 84 h and 96 h). The kinetics curve proved the significantly strong linear association between pigment production and bacterial growth (biomass production) (Figure 7). The specific rate the pigment production was calculated to be 1.25 × 10^−4^/h. The overall yield of the purified pigment was deduced to be 3.68%. In the present study, the obtained yield was higher than that of many other reported bacterial pigments. For example, the yield of an intracellular yellow pigment collected from *Vibrio owensii* was reported to be 1.14% [34]. The yield of prodigiosin from *Serratia marcescens* was also lower using freeze–thawing and homogenization [35].

### 3.8. Nano-Pigment Synthesis and Characterization

**Figure 8:**
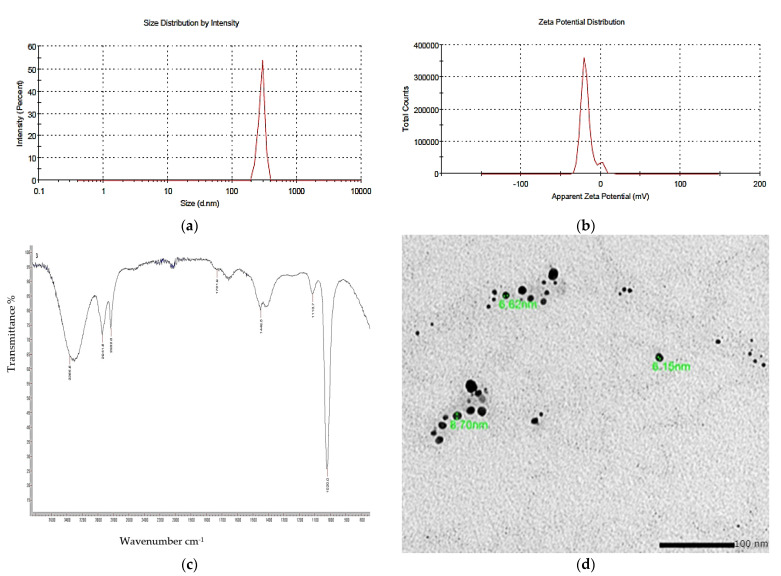
Physicochemical properties of the nano-echinenone of *Micrococcus lylae* MW407006 (**a**) Zeta size and (**b**) potential, (**c**) FTIR spectra, and (**d**) TEM micrograph.

The synthesized nano-echinenone using the ball-milling technique was characterized using the zeta potential, PDI, FTIR, and a transmission electron microscope (TEM) for the determination of the nanoparticles’ shape, size, homogeneity, and stability. It was found that the newly synthesized nano-echinenone had a circular shape with an average size of 11.3 ± 4 nm (Figure 8). The zeta potential and PDI were +30.4 mv and 0.3, respectively, which proved the nano-echinenone’s stability. 

### 3.9. Biological Activity of the Synthesized Nano-Echinenone

#### 3.9.1. Antimicrobial Effect of the Nano-Echinenone

**Figure 9:**
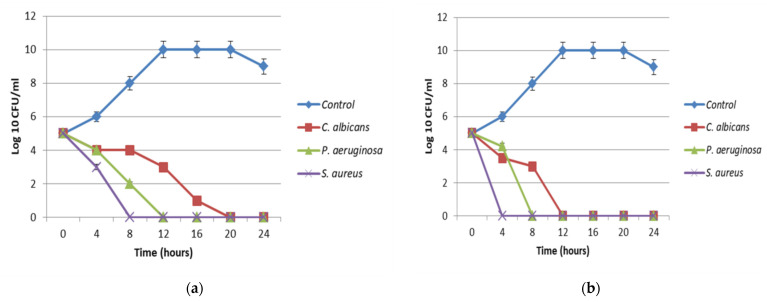
Microbial time–kill curve of the (**a**) optimized pigment and (**b**) the synthesized nano-echinenone against *Staphylococcus aureus, Pseudomonas aeuroginosa*, and *Candida albicans*.

**Figure 10:**
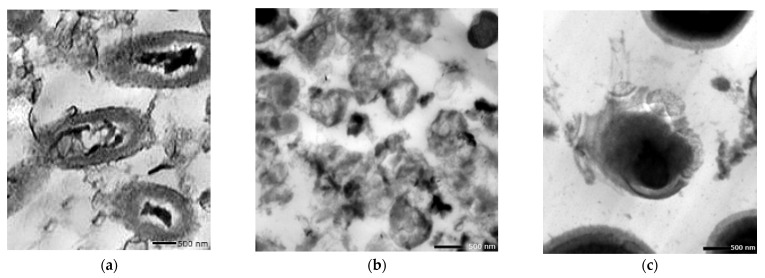
Transmission electron microscopic (TEM) studies of nano-echinenone-treated (**a**) *Pseudomonas aeruginosa*, (**b**) *Staphylococcus aureus,* and (**c**) *Candida albicans* cells.

The antimicrobial activity of the synthesized nano-echinenone was evaluated using disc diffusion, MIC, MBC, and the time–kill curve. The data revealed that the synthesized nano-echinenone had greater antimicrobial activity than the crude pigment extract, with inhibition zone diameters of 25, 20, and 20 mm against *Staphylococcus aureus, Pseudomonas aeruginosa,* and *Candida albicans,* respectively. On the other hand, the MIC values were reduced by almost 62.5, 44, and 31% against *Pseudomonas aeruginosa, Staphylococcus aureus,* and *Candida albicans,* respectively (Table 5). The data of the microbial lethality curve revealed the complete eradication of microbial growth after 4, 8, and 10 h against *Staphylococcus aureus, Pseudomonas aeruginosa*, and *Candida albicans*, respectively (Figure 9b), compared with the crude pigment, which inhibited the microbial growth after 8, 12, and 20 h against *Staphylococcus aureus, Pseudomonas aeruginosa*, and *Candida albicans*, respectively (Figure 9a). Moreover, the transmission electron microscopy study proved the antimicrobial effect of the synthesized nano-echinenone, and the treated cells became ghost cells (Figure 10).

The promising antimicrobial activity of the synthesized nano-echinenone may pave the way against opportunistic and antibiotic-resistant pathogens. The World Health Organization (WHO) released a list of 12 bacteria that were considered to be major concerns to human health due to their emerging antibiotic resistance, with *P. aeruginosa* being among the most virulent pathogens [36]. Novel drug development is not enough nowadays to combat drug-resistant microorganisms. Bacteria can produce melanins, carotenoids, flavins, monascins, quinones, and violaceins, which are known pigments with antibacterial properties [37]. *Streptomyces hygroscopicus*’ bioactive pigment showed potent antibacterial activity against vancomycin-resistant *S. aureus* (VRSA), MRSA, and extended-spectrum β-lactamase (ESBL) cultures of *E. coli* and *Klebsiella* spp. [38]. It was reported that the orange pigment of *B. megaterium* SU15 had antibacterial activity against *Salmonella Typhi* and *Escherichia coli*. It was also determined that the yellow pigment of *Streptomyces hygroscopicus* had activity against *Klebsiella* sp., VRSA, MRSA, and ESBL cultures of *E. coli* [38].

#### 3.9.2. Antioxidant Activity

One of the most important applications of natural products is using their antioxidant activity. The crude extracted pigment showed 87% DPPH activity, while that of the synthesized nano-echinenone was 96%, which proved the superior activity of the synthesized nanoparticles. Arulselvi et al. [39] reported that DPPH radicals were stoichiometrically decolorized by some efficient reducing compounds and antioxidants, e.g., cysteine, ascorbic acid, glutathione, and tocopherols. Sasidharan et al. [40] tested the antioxidant activity of eight bacterial pigments, and the DPPH-free radical scavenging activity (78%) was observed with the carotenoid-producing bacterium *Exiguobacterium* spp. Generally, carotenoids are potent antioxidants that can be used in vivo [39] and can be improved by using the nanoparticulate form of the potent pigments. Moreover, carotenoids play a major role in antioxidant and anticarcinogenic characteristics. The bioactive properties of these compounds are attributed to the carotenoid structures. Carotenoids were considered as ideal antioxidant substances due to the presence of the polyene chain that possesses several conjugated double bonds [41]. Despite the fact that cancer death rates have decreased continuously over the past two decades and about 1.7 million cancer cases were prevented, causing an overall drop of 23%, cancer is still one of the leading causes of mortality. Continued basic and clinical research is required to fight against this fatal disease [42]. Among all of the potential anticancer drugs from natural sources, bacterial pigments have been assessed less in the past, but the trend has been shifted toward them, with many recent studies in this regard, e.g., the antitumor properties of prodigiosin from *S. marcescens* have been studied [43]. 

#### 3.9.3. Cytotoxicity Assay against Human Cancer Cell Lines

**Figure 11:**
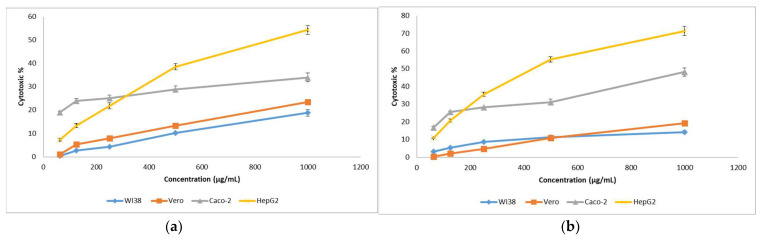
Cytotoxicity of *Micrococcus lylae* MW407006 echinenone (**a**) and nano-echinenone (**b**) on WI38, Vero, Caco-2, and Hep-G2.

In a trial to test the cytotoxic potency of echinenone and nano-echinenone, different cell lines of human tumors and normal cells were investigated. The inhibition of cell proliferation and induction of cell death were observed. Data revealed that the optimized extracted echinenone’s and nano-echinenone’s cytotoxic effects toward normal cells and cancer cells were directly proportional to the pigment concentration (Figure 11). The IC50 of the optimized extracted echinenone and nano-echinenone against normal cells, namely W138 and Vero models, were 2645 and 3521 µg/mL, respectively, and 2128 and 2604 µg/mL, respectively. The results indicated that nano-echinenone was safer than the crude pigment with higher IC50 toward both models. On the other hand, the efficacy of the synthesized nano-echinenone against cancer cells, namely Caco-2 and Hep-G2, proved its powerful effect compared with the crude pigment, which paves the way for its use as a therapy against colorectal and hepatocellular carcinoma (Table 6). The calculated selectivity index indicates that the nano-echinenone was much safer and more effective, especially against the Hep-G2 cells, where it had the highest selectivity index. Moreover, these data proved that nano-echinenone is a good candidate to be used as an anti-hepatocellular carcinoma agent. 

In accordance with the present results, β-carotene, an orange–yellow pigment isolated from *Rhodococcus maris,* reduced the risk of breast cancer [4]. Doxorubicin, a red pigment isolated from *Streptomyces peucetius* and *Streptomyces venezuelae*, proved itself as a promising anti-tumor compound [44]. In addition to these, deinoxanthin and quinone–anthracycline are unique, red-colored bioactive compounds isolated from *D. radiodurans* and *Streptosporangium* with strong anti-tumor therapeutic potential [45].

##### Determination of Cellular Reactive Oxygen Species (ROS)

**Figure 12:**
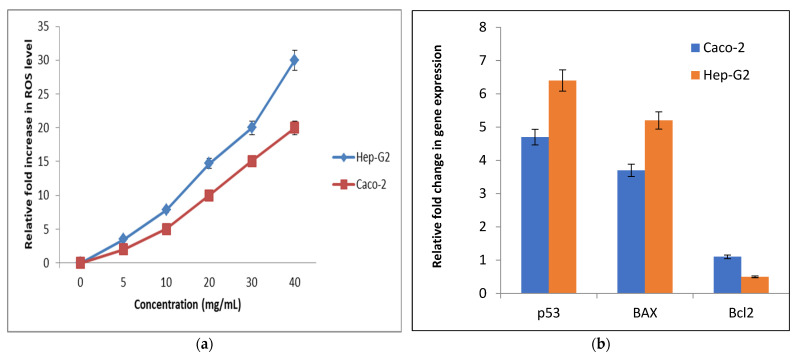
Prooxidant effect-mediated apoptotic activity of nano-echinenone human cancer cell lines (Caco-2 and HepG-2): (**a**) relative fold-increase in ROS level in human cancer cell lines after 72 h of incubation with serial concentrations of nano-echinenone; (**b**) relative changes in p53, BAX, and Bcl2 expression in IC_50_ of nano-echinenone-treated cancer cells.

The change in the redox status of the treated human cancer cells was detected based on the level of oxidized fluorescence DCF. Figure 12a illustrates that nano-echinenone caused a dose-dependent elevation in ROS in the studied cancer cell lines. At 20 mg/mL of nano-echinenone, the ROS level was increased by 10- and 14.7-fold in Caco-2 and Hep-G2 cells, respectively. 

##### Relative Changes in the Genetic Expression of Proapoptotic (p53 and BAX) and Anti-Apoptotic (Bcl2) Genes

The potential of nano-echinenone for apoptosis induction was studied using q-PCR, which assessed the proapoptotic and antiapoptotic genes’ expression changes. Figure 12b demonstrates the proapoptotic potency of nano-echinenone for p53 expression, which reached 4.7- and 6.4-fold in the treated Caco-2 and Hep-G2 cells, respectively. Moreover, BAX expression was increased by 3.7 and 5.2-fold in the treated Caco-2, and Hep-G2 cells, respectively, while Bcl2 (antiapoptotic gene) expression was down-regulated by 1.1- and 0.5-fold, respectively (Figure 12b, Table S4).

## 4. Conclusions

The data from the present investigation concluded that yellowish-orange pigment from *Micrococcus lylae* MW407006 showed potent pharmacological activities. The extracted pigment was identified as echinenone, and the carbon and nitrogen sources (linear levels) were the most significant factors that affected all of the investigated responses (biomass production, antimicrobial activity, and pigment production). The Spearman correlation coefficient indicated a significantly strong linear relationship between the investigated responses, specifically between the pigment and biomass production. Moreover, the kinetic analysis showed that the final yield of the purified pigment was 3.68%. Novel nano-echinenone was synthesized through the ball-milling technique, which showed higher antimicrobial activity than the crude one, with potent anti-hepatocellular carcinoma activity through cell proliferation inhibition and the induction of cell death. The significantly high selectivity index of the synthesized nano-echinenone proved its safety and paves the way for its use in the pharmaceutical industry.

## Figures and Tables

**Table 1:** Coded levels of the central composite analysis.

Parameter	Coded Levels				
	−2	−1	0	1	2
Carbon source % (glucose)	0.1	0.3	0.5	0.7	0.9
Nitrogen source % (Aparagine)	0.1	0.2	0.3	0.4	0.5
Incubation time (days)	2.0	3.0	4.0	5.0	6.0
pH	5.0	6.0	7.0	8.0	9.0
Temperature (°C)	15.0	20.0	25.0	30.0	35.0

**Table 2:** Screening for the antimicrobial activity of the extracted pigments.

Tested Pathogens		Inhibition Zone Diameter (mm) ± SD					
	Pigment Producer	Isolate 1	Isolate 2	Isolate 3	Isolate 4	Isolate 5	Isolate 6
*Pseudomonas aeruginosa*		8.0 ± 0.21	8.0 ± 0.26	12.0 ± 0.2	8.0 ± 0.32	7.0 ± 0.1	9.0 ± 0.21
*Acinetobacter baumannii*		8.0 ± 0.3	7.0 ± 0.21	10.0 ± 0.1	10.0 ± 0.25	0.0 ± 0	9.0 ± 0.19
MRSA		8.0 ± 0.26	8.0 ± 0.13	11.0 ± 0.12	8.0 ± 0.22	7.0 ± 0	10.0 ± 0.25
*Klebsiella pneumoniae*		9.0 ± 0.1	0.0 ± 0	10.0 ± 0.41	8.0 ± 0.33	0.0 ± 0.12	10.0 ± 0.27
*Proteus vulgaris*		9.0 ± 0.2	7.0 ± 0.5	10.0 ± 0.35	7.0 ± 0.22	0.0 ± 0	10.0 ± 0.27
*Listeria monocytogenes*		7.0 ± 0.2	8.0 ± 0.01	9.0 ± 0.25	9.0 ± 0.35	7.0 ± 0.12	9.0 ± 0.1
*Escherichia coli*		7.0 ± 0.2	8.0 ± 0.29	9.0 ± 0.3	8.0 ± 0.1	7.0 ± 0.11	9.0 ± 0.15
*Salmonella Typhi*		7.0 ± 0.3	8.0 ± 0.3	9.0 ± 0.41	8.0 ± 0.4	7.0 ± 0.11	9.0 ± 0.2
*Candida albicans*		8.0 ± 0.4	0.0 ± 0	10.0 ± 0.42	8.0 ± 0.5	0.0 ± 0	9.0 ± 0.2
*Staphylococcus aureus*		9.0 ± 0.45	8.0 ± 0.1	12.0 ± 0.26	0.0 ± 0	8.0 ± 0.25	10.0 ± 0.27

SD: standard deviation.

**Table 3:** Optimization of the nutritional and environmental factors using central composite design.

Trials	Time	Temp	pH	Carbon Source	Nitrogen Source	Inhibition Zone Diameter (mm) (R1)			Biomass Production Concentration (g/L) (R2)	Pigment Production Concentration (g/L) (R3)
						*Pseudomonas aeruginosa*	*Staphylococcus aureus*	*Candida albicans*		
**1**	**−1**	**1**	**1**	**−1**	**−1**	**6.0 ± 0**	6.0 ± 0	6.0 ± 0.1	2.6 ± 0.1	0.1 ± 0.21
2	1	1	1	1	−1	7.0 ± 0.1	7.5 ± 0.12	6.5 ± 0.12	3.2 ± 0.2	0.2 ± 0.32
3	−1	1	−1	−1	1	11.5 ± 0.2	12.5 ± 0.2	11.5 ± 0.2	3.8 ± 0.21	0.65 ± 0.15
4	−1	−1	1	−1	−1	9.0 ± 0.2	10.0 ± 0.25	8.0 ± 0.24	3.4 ± 0.21	0.4 ± 0.21
5	1	−1	−1	1	1	10.0 ± 0.35	10.0 ± 0.41	10.0 ± 0.2	3.5 ± 0.1	0.5 ± 0.11
6	1	1	1	−1	1	10.0 ± 0.32	10.0 ± 0.22	10.0 ± 0.41	3.5 ± 0.32	0.5 ± 0.13
7	1	−1	−1	1	−1	9.0 ± 0.26	10.0 ± 0.21	8.0 ± 0.21	3.4 ± 0.35	0.4 ± 0.11
8	1	−1	1	1	−1	9.0 ± 0.24	10.0 ± 0.25	8.0 ± 0.22	3.4 ± 0.34	0.4 ± 0.05
9	−1	−1	−1	1	−1	7.0 ± 0.12	7.5 ± 0.01	6.5 ± 0	3.2 ± 0.32	0.2 ± 0.05
10	1	1	1	−1	−1	6.0 ± 0	6.0 ± 0	6.0 ± 0	2.7 ± 0.25	0.1 ± 0.01
11	−1	−1	1	1	1	8.0 ± 0.21	9.0 ± 0.21	8.0 ± 0.22	3.34 ± 0.21	0.3 ± 0.1
12	1	1	−1	1	1	10.5 ± 0.3	11.0 ± 0.2	10.0 ± 0.41	3.56 ± 0.29	0.55 ± 0.1
13	1	1	−1	1	−1	10.0 ± 0.25	10.0 ± 0.24	9.0 ± 0.31	3.5 ± 0.42	0.5 ± 0.04
14	−1	1	−1	1	1	9.0 ± 0.21	9.0 ± 0.14	8.0 ± 0.56	3.4 ± 0.22	0.4 ± 0.12
15	−1	−1	−1	−1	−1	6.0 ± 0.1	6.0 ± 0	6.0 ± 0	2.6 ± 0.33	0.1 ± 0.03
16	0	0	0	0	0	11.9 ± 0.26	12.0 ± 0.45	11.5 ± 0.2	3.9 ± 0.36	0.69 ± 0.21
17	0	0	0	0	−2.37841	0.0 ± 0	0.0 ± 0	0.0 ± 0	0.1 ± 0.01	0.07 ± 0.01
18	1	1	−1	−1	1	9.0 ± 0.25	10.0 ± 0.2	9.0 ± 0.15	3.4 ± 0.31	0.4 ± 0.56
19	0	0	0	−2.37841	0	0.0 ±0	0.0 ± 0	0.0 ± 0	0.1 ± 0	0.07 ± 0
20	0	0	0	0	2.37841	15.4 ± 0.2	20.0 ± 0.24	9.6 ± 0.47	4.12 ± 0.12	0.8 ± 0.1
21	0	0	0	0	0	11.9 ± 0.15	12.0 ± 0.25	11.0 ± 0.21	3.9 ± 0.21	0.69 ± 0.32
22	−2.37841	0	0	0	0	0.0 ±0	0.0 ± 0	0.0 ± 0	0.1 ± 0.01	0.07 ± 0
23	1	−1	1	1	1	11.0 ± 0.45	11.0 ± 0.54	10.0 ± 0.2	3.6 ± 0.21	0.6 ± 0.01
24	−1	−1	−1	−1	1	11.1 ±0.49	11.5 ± 0.52	10.0 ± 0.21	3.6 ± 0.21	0.6 ± 0.01
25	0	0	−2.37841	0	0	8.0 ± 0.51	8.0 ± 0.25	8.0 ± 0.19	3.34 ± 0.21	0.3 ± 0.01
26	0	2.37841	0	0	0	6.8 ± 0.01	7.0 ± 0.32	5.0 ± 0	3.0 ± 0.25	0.18 ± 0
27	−1	1	−1	1	−1	8.0 ± 0.17	9.0 ± 0.21	7.0± 0.1	3.34 ± 0.35	0.3 ± 0.03
28	0	0	0	0	0	11.9 ± 0.14	12.0 ± 0.28	11.0 ± 0.45	3.9 ±0.64	0.69 ± 0.35
29	0	0	0	0	0	11.9± 0.14	12.0 ± 0.28	10.0 ± 0.15	3.9 ± 0.63	0.69 ± 0.32
30	1	−1	1	−1	−1	11.0 ±0.45	12.0 ± 0.31	9.0 ± 0.32	3.6 ± 0.25	0.6 ± 0.01
31	0	0	0	0	0	11.9 ± 0.14	12.5 ± 0.25	11.0 ± 0.56	3.9 ± 0.21	0.69 ± 0
32	1	−1	1	−1	1	11.0 ± 0.45	11.0 ± 0.21	10.0 ± 0.48	3.6 ± 0.32	0.6 ± 0
33	0	0	0	0	0	11.9 ± 0.15	12.4 ± 0.35	9.0 ± 0.37	3.9 ± 0.41	0.69 ± 0.1
34	−1	1	1	−1	1	8.0 ± 0.21	8.0 ± 0.24	8.0 ± 0.32	3.34 ± 0.65	0.3 ± 0
35	−1	1	1	1	1	8.0 ± 0.22	8.0 ± 0.21	8.0 ± 0.31	3.34 ± 0.45	0.3 ± 0.02
36	−1	−1	1	−1	1	8.0 ± 0.22	8.0 ± 0.45	8.0 ± 0.48	2.8 ± 0.21	0.14 ± 0
37	1	−1	−1	−1	−1	10.0 ± 0.31	10.0 ± 0.32	8.0 ± 0.5	3.34 ± 0.24	0.3 ± 0.02
38	1	1	−1	−1	−1	11.0 ± 0.34	11.0 ± 0.34	10.0 ± 0.3	3.5 ± 0.64	0.5 ± 0
39	1	−1	−1	−1	1	11.7 ± 0.32	11.9 ± 0.45	9.0 ± 0.25	3.6 ± 0.49	0.6 ± 0
40	0	−2.37841	0	0	0	0.0 ± 0	0.0 ± 0	0.0 ± 0	3.85 ± 0.25	0.67 ± 0
41	−1	−1	−1	1	1	11.7 ± 0.11	11.8 ± 0.24	9.4 ± 0.35	3.85 ± 0.32	0.67 ± 0.1
42	−1	1	−1	−1	−1	8.0 ± 0.23	8.5 ± 0.54	7.0 ± 0.12	3.34 ± 0.32	0.3 ± 0
43	0	0	2.37841	0	0	6.0 ± 0.02	6.0 ± 0.24	6.0 ± 0	2.6 ± 0.23	0.1 ± 0
44	−1	1	1	1	−1	9.0 ± 0.42	8.3 ± 0.51	7.6 ± 0.1	3.4 ± 0.21	0.4 ± 0.1
45	0	0	0	2.37841	0	11.9 ± 0.26	12.5 ± 0.26	11.2 ± 0.47	3.9 ± 0.36	0.69 ± 0.25
46	0	0	0	0	0	11.9 ± 0.15	12.0 ± 0.54	11.0 ± 0.42	3.9 ± 0.25	0.69 ± 0
47	2.37841	0	0	0	0	0.0 ± 0	0.0 ± 0	0.0 ± 0	0.1 ± 0	0.07 ± 0
48	−1	−1	1	1	−1	10.0 ± 0.2	10.0 ± 0.21	8.0 ± 0.48	3.5 ± 0.21	0.5 ±0.01
49	1	1	1	1	1	11.5 ± 0.32	11.9 ± 0.32	11.0 ± 0.35	3.8 ± 0.35	0.65 ± 0.1
50	0	0	0	0	0	11.9 ± 0.25	12.0 ± 0.61	11.0 ± 0.34	3.9 ± 0.32	0.69 ± 0.12

**Table 4:** MIC, MBC, and MIC indexes of the optimized crude pigment against the tested pathogens.

Tested Pathogens	Optimized Pigment of ***Micrococcus lylae*** MW407006		
	MIC (μg/mL)	MBC (μg/mL)	MIC Index
***Pseudomonas aeruginosa***	32.0	256.0	8.0
***Staphylococcus aureus***	16.0	128.0	8.0
***Candida albicans***	64.0	512.0	8.0

IZ, inhibition zone; MIC, minimum inhibitory concentration; MBC, minimum bactericidal concentration.

**Table 5:** Antimicrobial effects of *Micrococcus lylae* MW407006 nano-pigment.

Tested Pathogen	Antimicrobial Effect			
	IZ (mm)	MIC (μg/mL)	MBC (μg/mL)	MIC Index
***Pseudomonas aeruginosa***	20.0 ± 0.17	20.0	150.0	7.5
***Staphylococcus aureus***	25.0 ± 0.42	7.0	100.0	14.2
***Candida albicans***	20.0 ± 0.09	20.0	150.0	7.5

IZ, inhibition zone; MIC, minimum inhibitory concentration; MBC, minimum bactericidal concentration.

**Table 6:** IC50 and the selectivity index of *Micrococcus lylae* MW407006 echinenone and nano-echinenone against Caco-2 and HEP-G2 after 72 h.

Sample	IC 50 (µg/mL)				Caco-2 Selectivity Index with		HEPG2 Selectivity Index with	
	WI38	Vero	Caco-2	HepG2	Vero	WI38	Vero	WI38
Echinenone	2645	2128	1496.4	920.8	1.422	1.768	2.311	2.87
Nano-echinenone	3521	2604	1537	450.1	1.694	2.29	5.785	7.822

## Data Availability

The datasets analyzed during the current study are available from the National Center for Biotechnology Information (NCBI) database. https://www.ncbi.nlm.nih.gov/nucleotide/MW407006.1?report=genbank&log$=nuclalign&blast_rank=1&RID=9XYVUSS0016&from=1&to=650 (accessed on 24 December 2020).

## Supplementary Materials

The following supporting information can be downloaded at: https://www.mdpi.com/article/10.3390/biology11081171/s1, Figure S1: ^1^H-NMR spectrum of the extracted pigment (echinenone).; Table S1: Susceptibility profiles of the tested pathogens to some commonly known antibiotics; Table S2: ANOVA study of the experimental output; Table S3: Fitting Statistics of the RSM design.
