# Supplementary material for: Micrococcus lylae MW407006 Pigment: Production, Optimization, Nano-Pigment Synthesis, and Biological Activities
Source: Biology (Basel). 2022 Aug 4;11(8):1171. doi: 10.3390/biology11081171 (PMC9405233; doi:10.3390/biology11081171)
Supplement: Supplementary file 1 [file biology-11-01171-s001.zip › biology-1810048-supplementary.pdf]

Micrococcus lylae MW407006 Pigment: Production, Optimization, Nano-Pigment Synthesis, and Biological Activities

Yahya H. Shahin [1,2], Bassma H. Elwakil [1,*], Doaa A. Ghareeb [3] and Zakia A. Olama [2]

1 Department of Medical Laboratory Technology, Faculty of Applied Health Sciences Technology, Pharos University in Alexandria, Alexandria 21648, Egypt; yahya.hamdy@pua.edu.eg
2 Department of Botany and Microbiology, Faculty of Science, Alexandria University, Alexandria 21526, Egypt; zakiaolama52@gmail.com
3 Biological Screening and Preclinical Trial Lab, Biochemistry Department, Faculty of Science, Alexandria University, Alexandria 21526, Egypt; d.ghareeb@yahoo.com
* Correspondence: bassma.hassan@pua.edu.eg

Supplementary data

Table S1. Susceptibility profiles of the tested pathogens to some commonly known antibiotics

Tested pathogens	Ampicillin		Cefazolin		Amikacin		Pipracillin		Colistin		Cefoxitin		Clotrimazole	
	IZ	MIC	IZ	MIC	IZ	MIC	IZ	MIC	IZ	MIC	IZ	MIC	IZ	MIC
Pseudomonas aeruginosa	R	R	R	R	10.0	20.0	10.0	21.0	R	R	11.0	20.0	--	--
Acinetobacter baumannii	R	R	R	R	**11.0**	21.0	9.0	22.0	R	R	10.0	23.3	--	--
**MRSA**	R	R	R	R	9.0	22.0	14.0	20.0	R	R	R	R	--	--
Klebsiella pneumoniae	R	R	R	R	**10.0**	25.0	9.0	23.5	R	R	9.0	21.5	--	--
Proteus vulgaris	R	R	R	R	**10.0**	23.0	11.0	25.0	R	R	8.0	24.0	--	--

Listeria monocytogenes	R	R	R	R	9.0	26.0	9.0	24.5	R	R	12.0	26.0	--	--
E. coli	R	R	R	R	8.0	29.0	7.0	27	R	R	13.0	25.0	--	--
Salmonella Typhi	R	R	R	R	7.0	28.0	9.0	25.5	R	R	11.0	24.3	--	--
Candida albicans	R	R	R	R	**11.0**	28.0	8.0	26.8	R	R	9.0	23.2	6.0	1500.0
Staphylococcus aureus	R	R	R	R	9.0	28.0	9.0	26.1	R	R	10.0	29.0	--	--

IZ, inhibition zone (mm); MIC, minimum inhibitory concentration (μg/mL); --, not applicable

Table S2. ANOVA study of the experimental output

Source	Antimicrobial activity*			Biomass production **(g/L)** *			Pigment production **(g/L)** *		
	Sum of squares	F-value	p-value	Sum of squares	F-value	P-value	Sum of Squares	F-value	p-value
**Model**	354.06	2.53	0.0112	28.02	1.98	0.0457	1.73	3.38	0.0014
A—Time	6.99	0.9979	0.3261	0.0878	0.1240	0.7272	0.0699	2.73	0.1090
B—Temp.	8.56	1.22	0.2781	0.2122	0.2998	0.5882	0.0856	3.35	0.0776
C—pH	4.24	0.6058	0.4427	0.2320	0.3278	0.5714	0.0424	1.66	0.2078
D—carbon source	28.45	4.06	0.0532	3.35	4.73	0.0378	0.1072	4.19	0.0498
E—Nitrogen source	86.55	12.36	0.0015	4.01	5.66	0.0242	0.4065	15.90	0.0004
AB	0.6050	0.0864	0.7709	0.0751	0.1061	0.7470	0.0061	0.2367	0.6303
AC	1.44	0.2063	0.6531	0.0621	0.0878	0.7691	0.0145	0.5652	0.4582
AD	0.2450	0.0350	0.8529	0.0185	0.0262	0.8726	0.0025	0.0958	0.7591
AE	0.3613	0.0516	0.8219	0.0102	0.0143	0.9055	0.0036	0.1413	0.7097
BC	4.65	0.6640	0.4218	0.0914	0.1291	0.7220	0.0465	1.82	0.1878
BD	0.1513	0.0216	0.8842	0.0081	0.0115	0.9154	0.0015	0.0592	0.8095
BE	0.1800	0.0257	0.8738	0.0158	0.0223	0.8824	0.0018	0.0704	0.7926
CD	0.9113	0.1301	0.7210	0.0358	0.0505	0.8237	0.0091	0.3564	0.5551

CE	3.65	0.5204	0.4765	0.0102	0.0143	0.9055	0.0365	1.43	0.2421
DE	0.3200	0.0457	0.8322	0.0158	0.0223	0.8824	0.0032	0.1252	0.7261
A^2	184.03	26.27	< 0.0001	16.80	23.74	< 0.0001	0.5829	22.80	< 0.0001
B^2	1.89	0.2704	0.6070	0.0799	0.1128	0.7394	0.0874	3.42	0.0746
C^2	18.85	2.69	0.1117	0.1005	0.1420	0.7091	0.3507	13.72	0.0009
D^2	32.77	4.68	0.0389	2.54	3.60	0.0679	0.1260	4.93	0.0344
E^2	11.69	1.67	0.2066	2.10	2.97	0.0954	0.0798	3.12	0.0878

*, the model is significant

Table S3. Fitting statistics of the RSM design

Antimicrobial activity				Biomass production **(g/L)**				Pigment production **(g/L)**			
**Std. Dev.**	2.65	**R^2**	0.6354	**Std. Dev.**	0.8413	**R^2**	0.5772	**Std. Dev.**	0.1599	**R^2**	0.6999
**Mean**	8.91	**Adjusted R^2**	0.3840	**Mean**	3.20	**Adjusted R^2**	0.2856	**Mean**	0.4320	**Adjusted R^2**	0.4930
**C.V. %**	29.70	**Predicted R^2**	-0.5805	**C.V. %**	26.28	**Predicted R^2**	-0.9078	**C.V. %**	37.01	**Predicted R^2**	-0.1374
		**Adeq Precision**	7.0064			**Adeq Precision**	6.6002			**Adeq Precision**	6.6682

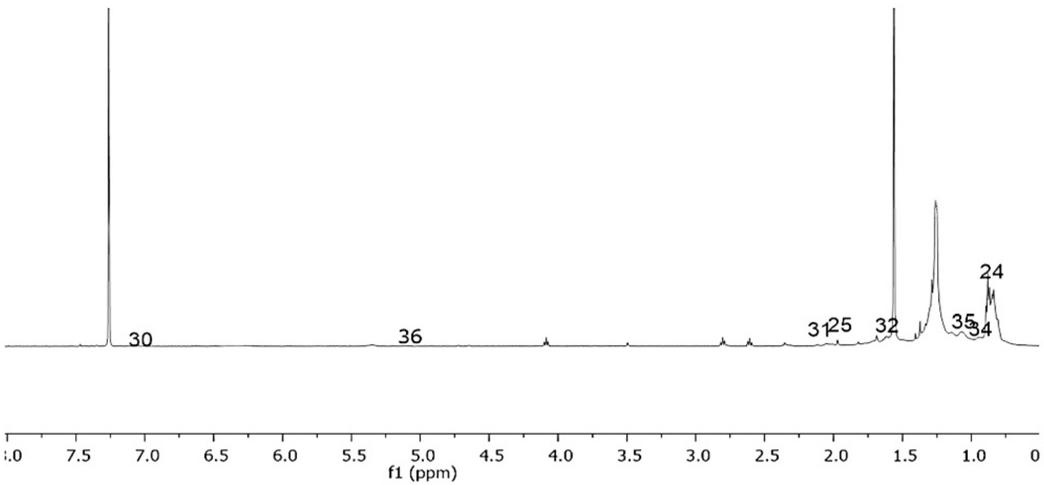

Figure S1. ^{1}H-NMR spectrum of the extracted pigment (echinenone).
